# Serum albumin concentrations are associated with disease severity and outcomes in coronavirus 19 disease (COVID-19): a systematic review and meta-analysis

**DOI:** 10.1007/s10238-021-00686-z

**Published:** 2021-01-28

**Authors:** Panagiotis Paliogiannis, Arduino Aleksander Mangoni, Michela Cangemi, Alessandro Giuseppe Fois, Ciriaco Carru, Angelo Zinellu

**Affiliations:** 1 Department of Medical, Surgical and Experimental Sciences, University of Sassari, Viale San Pietro 43, 07100 Sassari, Italy; 2 Discipline of Clinical Pharmacology, College of Medicine and Public Health, Flinders Medical Centre, Flinders University, Adelaide, Australia; 3 Department of Biomedical Sciences, University of Sassari, Viale San Pietro 43, 07100 Sassari, Italy

**Keywords:** Albumin, Coronavirus 2, COVID-19, SARS

## Abstract

Coronavirus disease 2019 (COVID-19), an infectious disease caused by the severe acute respiratory syndrome coronavirus 2 (SARS-CoV-2), is responsible for the most threatening pandemic in modern history. The aim of this systematic review and meta-analysis was to investigate the associations between serum albumin concentrations and COVID-19 disease severity and adverse outcomes. A systematic literature search was conducted in PubMed, from inception to October 30, 2020. Sixty-seven studies in 19,760 COVID-19 patients (6141 with severe disease or poor outcome) were selected for analysis. Pooled results showed that serum albumin concentrations were significantly lower in patients with severe disease or poor outcome (standard mean difference, SMD: − 0.99 g/L; 95% CI, − 1.11 to − 0.88, *p* < 0.001). In multivariate meta-regression analysis, age (*t* =  − 2.13, *p* = 0.043), publication geographic area (*t* = 2.16, *p* = 0.040), white blood cell count (*t* =  − 2.77, *p* = 0.008) and C-reactive protein (*t* =  − 2.43, *p* = 0.019) were significant contributors of between-study variance. Therefore, lower serum albumin concentrations are significantly associated with disease severity and adverse outcomes in COVID-19 patients. The assessment of serum albumin concentrations might assist with early risk stratification and selection of appropriate care pathways in this group.

## Introduction

Coronaviruses are a large family of enveloped positive-sense RNA viruses known to cause clinical symptoms ranging from the common cold to severe respiratory infections, such as the severe acute respiratory syndrome (SARS) and the Middle East respiratory syndrome (MERS). The latter, caused by SARS-CoV and MERS-CoV, respectively, have caused recent epidemics with mortality rates ranging from 10 to 37% [1–4]. Coronavirus disease 2019 (COVID-19) is a recently identified infectious disease caused by the novel severe acute respiratory syndrome coronavirus 2 (SARS-CoV-2) [5]. COVID-19 represents the most threatening pandemic outbreak in modern history, affecting some fifty million people and causing more than 1.2 million deaths globally as of November 12, 2020. Actions for the containment of the disease have included different degrees of lockdown strategies in many countries, generating unpredictable economic and social consequences. The spectrum of COVID-19 illness ranges from being asymptomatic or experiencing mild symptoms to important clinical manifestations such as severe pneumonia, which can further progress to acute respiratory distress syndrome (ARDS), multiple organ failure and, potentially, death [6, 7]. An increased risk of severe disease and adverse outcomes has been observed in older adults and patients of any age with comorbidities such as coronary heart disease, diabetes, respiratory disease and hypertension [6, 8]. There are ongoing efforts to better understand the pathophysiology, presentation and clinical outcomes of the disease, including the identification of biomarkers for diagnosis, risk stratification, disease monitoring and prognosis. Early studies in COVID-19 patients have reported alterations in routine laboratory tests, particularly white blood cell count, neutrophils, lymphocytes, platelets, alanine aminotransferase, aspartate aminotransferase, D-dimer, total bilirubin and creatinine [8–11]. Reductions in serum albumin concentrations have also been associated with disease severity [12, 13]. The aim of this present study was to appraise the available evidence regarding the associations between serum albumin concentrations, disease severity and adverse outcomes in COVID-19 patients.

## Materials and methods

### Search strategy, eligibility criteria and study selection

An electronic search was performed in Medline (PubMed interface) using the keywords “albumin” AND “coronavirus” OR “albumin” AND “COVID-19” from inception to October 30, 2020. The inclusion criteria were: (a) studies reporting continuous data on serum albumin concentrations in COVID-19 patients, (b) articles investigating COVID-19 patients with different disease severity or clinical outcomes, (c) articles in adult patients, (d) number of studied patients ≥ 10, (e) articles in English and (f) full-text article was available. Two investigators independently screened the abstracts to establish relevance. If relevant, the two investigators independently reviewed the full articles. Any disagreement between the reviewers was resolved by a third investigator. The reference list of the studies identified was also checked in order to identify additional studies. We used the Newcastle–Ottawa scale to assess the quality of each study [14]. The Newcastle–Ottawa scale evaluates the following components: cohort selection, cohort comparability on the basis of the design or analysis, how the exposure is determined and how the outcomes of interest are evaluated. Studies achieving a score of six or more were considered to be of high quality.

### Endpoint

The study endpoint was the pooled SMD of serum albumin concentrations between patients with low versus high severity or good versus poor outcomes. Disease severity was based on symptoms, disease progression (from moderate to severe grade or critical grade), ICU admission, intubation and ARDS onset, whereas outcome was based on survival vs. death during the study period.

### Statistical analysis

Standardized mean differences (SMD) were used to build forest plots of continuous data and to evaluate differences in serum albumin concentrations between COVID-19 patients with low versus high severity or good vs. poor outcomes. A *p*-value < 0.05 was considered statistically significant, and 95% confidence intervals (CIs) were reported. When required, the mean and standard deviation values were extrapolated from median and IQR as previously reported by Wan et al. [15] or median and range as reported by Hozo et al. [16]. Heterogeneity of SMD across studies was tested by using the Q statistic (significance level at *p* < 0.10). The *I*^2^ statistic, a quantitative measure of inconsistency across studies, was also calculated (*I*^2^ < 25%, no heterogeneity; *I*^2^ between 25 and 50%, moderate heterogeneity; *I*^2^ between 50 and 75%, large heterogeneity; and *I*^2^ > 75%, extreme heterogeneity). A random-effects model was used if heterogeneity was high. Sensitivity analysis was conducted to investigate the influence of individual studies on the overall risk estimate, by sequentially excluding one study at a time. To evaluate the presence of potential publication bias, the associations between study size and magnitude of effect were analysed by means of Begg’s adjusted rank correlation test and Egger’s regression asymmetry test at the *p* < 0.05 level of significance [17, 18]. Duval and Tweedie “trim and fill” procedure was performed to identify and correct for funnel plot asymmetry arising from publication bias [19]. Statistical analyses were performed using MedCalc for Windows, version 15.4 64 bit (MedCalc Software, Ostend, Belgium) and Stata 14 (STATA Corp., College Station, TX, USA).

## Results

### Electronic search results and characteristics of the included studies

**Fig. 1:**
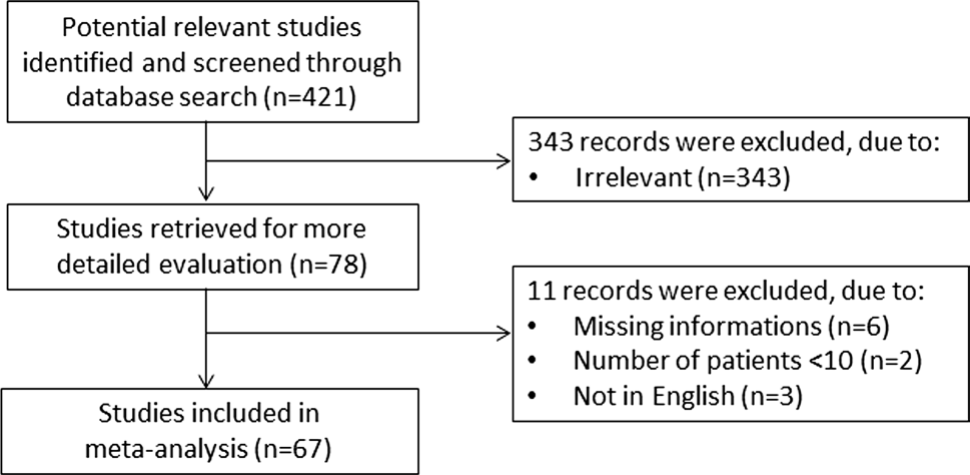
Flow chart illustrating the electronic search strategy and review

A flow chart describing the screening process is presented in Fig. 1. We initially identified 421 studies. A total of 343 studies were excluded after the first screening because they were either duplicates or irrelevant. After full-text review of the remaining 78 articles, a further 11 studies were excluded because they did not meet the inclusion criteria. Thus, 67 studies were included in the meta-analysis [20–86]. The characteristics of these studies, all published in 2020, are presented in Table 1. A total of 19,760 COVID-19 patients were studied, 13,628 (49% males, mean age 53 years) with low severity or favourable outcome and 6141 (58% males, mean age 65 years) with high severity or poor outcome. Three studies were prospective [29, 41, 52], 51 retrospective [20–28, 30–34, 36–39, 42–46, 48–51, 53, 54, 57–60, 62, 63, 65, 66, 69–76, 78, 80, 81, 83–86], while 13 did not specifically declare the study design [23, 35, 40, 47, 55, 56, 61, 64, 67, 68, 77, 79, 82]. Fifty-two studies (77.6%) were performed in China [22, 24–26, 28–31, 33–35, 37–43, 46–57, 59, 62–64, 68–85] while the remaining 15 were conducted in the rest of the world [20, 21, 23, 27, 32, 36, 44, 45, 58, 60, 61, 65–67, 86]. Endpoints included disease severity based on current clinical guidelines (31 studies, 46%) [22, 24, 28, 32, 33, 35, 36, 38, 39, 44–47, 49–52, 54, 56, 58, 64, 66, 69, 72, 73, 75, 77, 79, 81–83], survival (19 studies, 28%) [20, 23, 26, 30, 31, 34, 42, 53, 59–62, 65, 67, 71, 76, 84–86], intensive care unit (ICU) admission (7 studies, 10%) [21, 27, 41, 48, 68, 70, 78] and other outcomes (10 studies, 15%) [25, 29, 37, 40, 43, 55, 57, 63, 74, 80]. Among the 67 retrieved studies, only Aloisio et al. [20] reported the lowest albumin concentrations throughout hospitalization, whereas all the remaining studies reported albumin concentrations measured within the first 24–48 h from admission.

**Table 1:** Summary of the studies in COVID-19 patients, according to disease severity or outcome, included in the meta-analysis

				Mild disease or good outcome				Severe disease or poor outcome			
First Author, Country, Reference	Study design	Outcome	NOS (stars)	*n*	Age (Years)	Gender (M/F)	Albumin (g/L, Mean ± SD)	*n*	Age (Years)	Gender (M/F)	Albumin (g/L, Mean ± SD)
Aloisio et al. Italy [20]	R	Survivor Non-Survivor	7	338	58	223/115	28.3 ± 5.2	89	73	70/19	20.3 ± 5.4
Batsug et al. Turkey [21]	R	ICU Non-ICU	8	145	43	81/64	47.5 ± 6.8	46	71	26/20	35.8 ± 7.8
Bi et al. China [22]	R	Severe Non-severe	7	91	44	51/40	41.3 ± 4.0	22	54	13/9	38.8 ± 4.5
Bonetti et al. Italy [23]	U	Survivor Non-Survivor	7	74	62	51/23	36.5 ± 4.2	70	78	45/25	34.1 ± 3.1
Cao et al. China [24]	R	Severe Non-severe	8	53	44	22/31	36.8 ± 4.3	27	71	16/11	32.8 ± 6.5
Cheng et al. China [25]	R	No progression Poor prognoses*	8	205	49	71/134	40.2 ± 5.9	251	60	140/111	37.9 ± 6.3
Cheng et al. China [26]	R	Survivor Non-Survivor	8	53	54	29/24	39.2 ± 3.7	36	69	20/16	34.0 ± 5.2
de la Rica et al. Spain [27]	R	ICU Non-ICU	7	27	66	18/9	39.2 ± 4.2	21	66	14/7	29.0 ± /5.2
Deng et al. China [28]	R	Severe Non-severe	8	53	35	24/29	42.0 ± 3.2	12	33	12/0	40.9 ± 3.6
Feng et al. China [29]	P	Good outcome Poor outcome**	8	94	63	58/36	35.6 ± 5.5	20	69	13/7	29.9 ± 4.4
Gan et al. China [30]	R	Survivor Non-Survivor	7	56	62	30/26	34.9 ± 4.4	39	70	28/11	30.5 ± 4.5
Gao et al. China [31]	R	Survivor Non-Survivor	8	175	70	79/96	36.2 ± 5.1	35	74	22/13	30.8 ± 6.1
Ghweil et al. Egypt [32]	R	Severe Non-severe	7	36	56	28/8	29.0 ± 1.0	30	63	20/10	25.0 ± 2.0
Gong et al. China [33]	R	Severe Non-severe	7	161	45	72/89	39.7 ± 4.3	28	64	16/12	34.2 ± 5.1
Guo et al. China [34]	R	Survivor Non-Survivor	8	43	60	22/21	29.4 ± 1.4	31	68	21/10	26.5 ± 3.3
He et al. China [35]	U	Severe Non-severe	7	32	42	15/17	41 ± 5.4	21	57	13/8	31.7 ± 4.0
Hirashima et al. Japan [36]	R	Severe Non-severe	7	45	42	27/18	39.5 ± 4.5	16	63	13/3	31.0 ± 4.0
How et al. China [37]	R	Progression Improvement***	8	84	47	34/50	36.5 ± 5.2	17	72	10/7	32.3 ± 7.2
Hu et al. China [38]	R	Severe Non-severe	8	19	43	11/8	41.6 ± 4.3	21	63	13/8	35.6 ± 5.1
Hu et al. China [39]	R	Severe Non-severe	8	130	63	58/72	31.4 ± 6.2	52	64	42/10	27.2 ± 5.7
Hua et al. China [40]	U	Ventilation^§^ No ventilation	7	204	67	108/96	32.7 ± 4.6	265	69	158/107	31.4 ± 6.1
Huang et al. China [41]	P	ICU Non-ICU	8	28	49	19/9	33.8 ± 4.9	13	49	11/2	28.4 ± 3.8
Huang et al. China [42]	R	Survivor Non-Survivor	7	283	53	149/134	37.6 ± 6.2	16	69	11/5	30.5 ± 4.0
Huang et al. China [43]	R	Critical or Dead Non-critical	8	2,008	60	943/1,065	36.7 ± 5.3	615	66	380/235	32.0 ± 4.6
Hundt et al. USA [44]	R	Severe Non-severe	8	1,175	63	574/601	34.0 ± 3.0	652	67	395/257	31.0 ± 2.7
Lee et al. Republic of Korea [45]	R	Severe Non-severe	8	557	52	155/402	41.2 ± 3.9	137	71	57/80	35.3 ± 5.0
Lei et al. China [46]	R	Severe Non-severe	8	50	62	22/28	35.5 ± 4.1	65	69	36/29	33.2 ± 4.9
Li et al. China [47]	U	Severe Non-severe	7	41	53	20/21	40.0 ± 3.9	24	56	15/9	35.0 ± 5.9
Li et al. China [48]	R	ICU Non-ICU	8	312	49	131/181	40.7 ± 3.7	211	62	119/92	36.3 ± 5.2
Li et al. China [49]	R	Severe Non-severe	7	45	50	24/21	40.4 ± 3.5	89	64	51/38	36.0 ± 6.0
Lian et al. China [50]	R	Severe Non-severe	8	140	66	62/78	38.4 ± 4.4	92	68	47/45	35.2 ± 5.6
Liang et al. China [51]	R	Severe Non-severe	8	1,459	48	816/643	33.9 ± 8.9	131	66	88/43	32.6 ± 8.9
Liu et al. China [52]	P	Severe Non-severe	7	44	41	21/23	44.0 ± 4.6	17	56	10/7	41.8 ± 6.8
Liu et al. China [53]	R	Survivor Non-Survivor	7	302	64	148/154	35.8 ± 5.2	34	74	21/13	27.6 ± 3.2
Liu et al. China [54]	R	Severe Non-severe	8	561	43	288/273	41.9 ± 5.7	64	60	41/23	38.4 ± 5.5
Liu et al. China [55]	U	Progression Improvement***	7	67	37	32/35	41.3 ± 4.6	11	66	7/4	36.6 ± 6.6
Ma et al. China [56]	U	Severe Non-severe	8	429	42	199/230	40.7 ± 5.2	94	50	35/59	38.9 ± 6.6
Mo et al. China [57]	R	Refractory General	8	70	46	31/39	39.0 ± 4.5	85	61	55/30	36.0 ± 6.0
Mori et al. Japan [58]	R	Severe Non-severe	8	23	69	13/10	34.7 ± 10.9	22	58	21/1	25.0 ± 8.7
Pan et al. China [59]	R	Survivor Non-Survivor	8	35	65	18/17	29.1 ± 4.2	89	69	67/22	28.1 ± 3.7
Pourabdollah Toutkaboni et al. Iran [60]	R	Survivor Non-Survivor	**7**	481	55	301/180	34.7 ± 4.1	89	64	68/21	30.0 ± 4.0
Recinella et al. Italy [61]	U	Survivor Non-Survivor	8	66	79	32/34	31.9 ± 5.7	43	86	22/21	27.0 ± 5.2
Sheng et al. China [62]	R	Survivor Non-Survivor	7	144	66	NR	37.5 ± 5.2	88	75	NR	31.1 ± 3.3
Shi et al. China [63]	R	ICUorDeath Non-ICU	7	51	58	27/24	36.2 ± 5.1	36	66	22/14	29.3 ± 5.1
Sun et al. China [64]	U	Severe Non-severe	7	44	42	25/19	40.2 ± 3.9	19	59	11/8	33.5 ± 5.4
Tsibouris et al. Greece [65]	R	Survivor Non-Survivor	7	45	NR	NR	33.0 ± 6.1	16	NR	NR	26.0 ± 5.3
Varim et al. Turkey [66]	R	Severe Non-severe	7	85	62	48/37	33.3 ± 5.4	59	69	31/28	29.8 ± 4.9
Violi et al. Italy [67]	U	Survivor Non-Survivor	8	255	66	148/107	33.9 ± 5.3	64	77	45/19	30.3 ± 5.1
Wang et al. China [68]	U	ICU Non-ICU	8	39	54	27/12	37.6 ± 4.0	46	65	18/28	33.2 ± 5.4
Wang et al. China [69]	R	Severe Non-severe	7	72	44	29/43	38.6 ± 2.3	71	65	44/27	32.0 ± 3.0
Wang et al. China [70]	R	ICU Non-ICU	8	14	66	11/3	35.0 ± 5.9	14	71	10/4	30.5 ± 4.3
Wang et al. China [71]	R	Survivor Non-Survivor	7	175	64	86/89	34.9 ± 4.7	24	70	16/8	31.6 ± 4.6
Wang et al. China [72]	R	Severe Non-severe	7	79	41	38/41	42.1 ± 5.1	26	59	18/8	37.7 ± 5.9
Wang et al. China [73]	R	Severe Non-severe	7	230	45	102/128	41.0 ± 4.4	45	61	26/19	34.3 ± 3.8
Wu et al. China [74]	R	ARDS Non-ARDS	7	117	48	68/49	33.7 ± 4.0	84	59	60/24	30.3 ± 4.7
Xue et al. China [75]	R	Severe Non-severe	7	56	61	30/26	34.8 ± 3.8	58	64	34/24	28.0 ± 7.3
Yao et al. China [76]	R	Survivor Non-Survivor	8	96	48	36/60	39.3 ± 3.6	12	63	7/5	31.1 ± 4.7
Yu et al. China [77]	U	Severe Non-severe	7	799	61	384/415	35.7 ± 4.7	864	66	454/410	34.5 ± 5.1
Zeng et al. China [78]	R	ICU Non-ICU	7	406	43	206/200	40.3 ± 5.4	55	60	33/22	35.6 ± 5.4
Zhang et al. China [79]	U	Severe Non-severe	7	56	43	24/32	37.5 ± 3.9	24	65	9/15	32.8 ± 5.4
Zhang et al. China [80]	R	Normal Abnormal^§§^	7	72	35	33/39	42.5 ± 4.7	573	47	295/278	41.0 ± 4.5
Zhang et al. China [81]	R	Severe Non-severe	7	84	44	29/55	40.4 ± 3.2	31	65	20/11	34.4 ± 4.1
Zheng et al. China [82]	U	Severe Non-severe	7	55	44	26/29	33.9 ± 9.4	13	61	10/3	32.5 ± 3.2
Zhou et al. China [83]	R	Severe Non-severe	8	95	35	38/57	42.4 ± 4.5	28	40	17/11	40.4 ± 4.8
Zhou et al. China [84]	R	Survivor Non-Survivor	8	137	52	56/81	33.5 ± 4.4	54	69	16/38	29.0 ± 3.6
Zhou et al. China [85]	R	Survivor Non-Survivor	7	51	73	31/20	33.6 ± 4.9	67	71	22/45	33.1 ± 5.0
Zinellu et al. Italy [86]	R	Survivor Non-Survivor	8	77	68	27/50	34.7 ± 5.3	28	80	8/20	32.3 ± 3.9

*ARDS* Acute respiratory distress syndrome; *ICU* intensive care unit; *Non-severe* Patients with mild or moderate disease; *NOS* Newcastle–Ottawa quality assessment scale for case–control studies; *NR* Not Reported; *P* prospective; *R* retrospective; *Severe* Patients with severe or critical disease; *U* undeclared.

^*^Poor prognosis refers to progression from moderate to severe grade, critical grade or death.

^**^Patients that were discharged, those with non-severe condition, and those not requiring mechanical ventilation were considered to have a good outcome. Patients requiring mechanical ventilation and those who died were considered to have a poor outcome.

^***^Progression group: clinically advanced types; patients admitted to ICU; death during hospitalization. Improvement group: clinical status remained unchanged or improved, and patients discharged from the hospital.

^§^Both invasive and non-invasive ventilation.

^§§^Normal or abnormal imaging findings.

### Meta-analysis

**Fig. 2:**
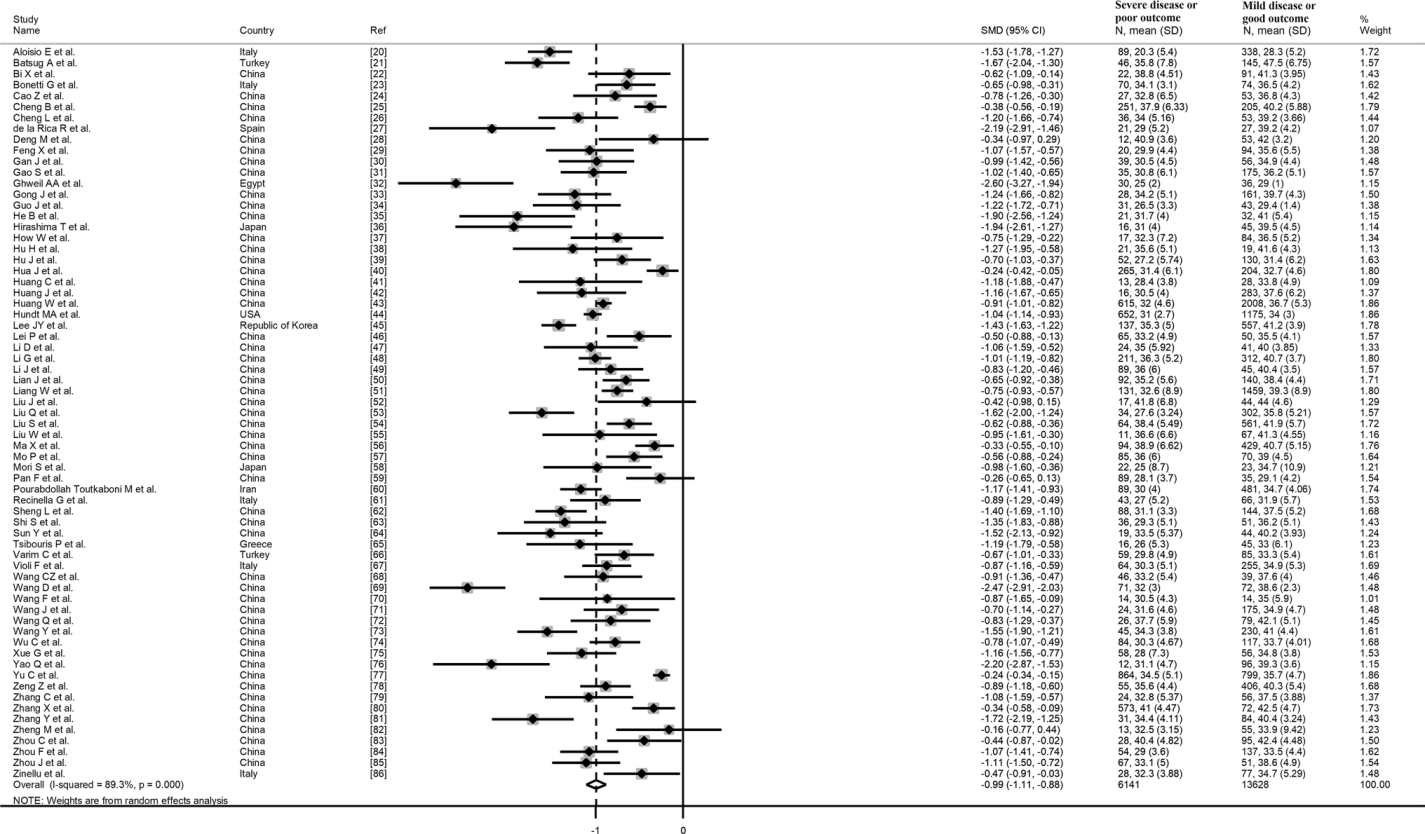
Forest plot of SMD differences of serum albumin concentrations between COVID-19 patients with low/high severity and good/poor outcome

**Fig. 3:**
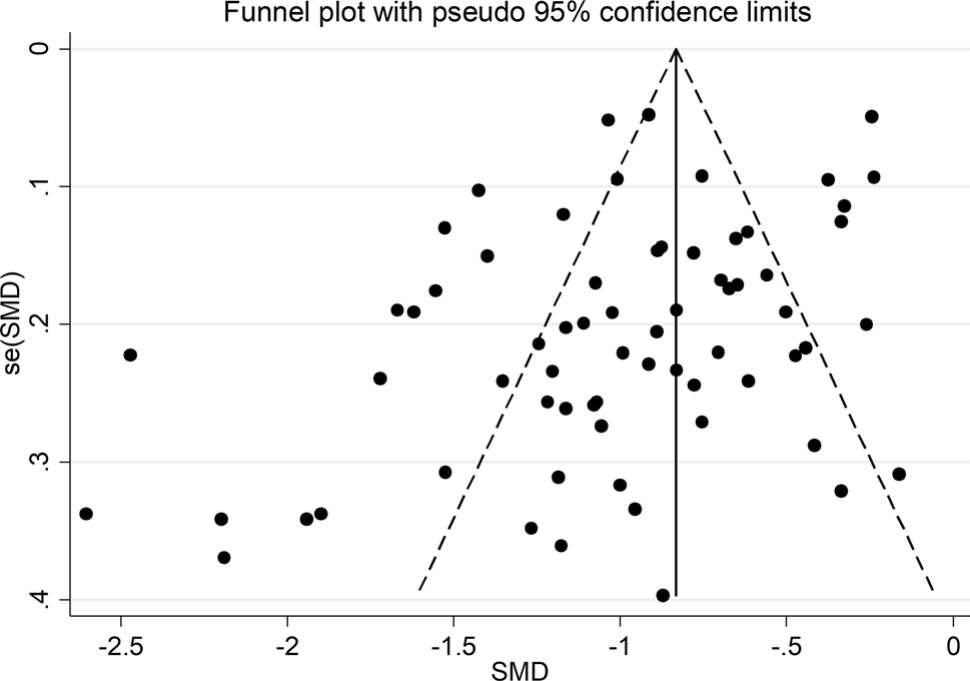
Funnel plot of studies examining albumin and severity and outcome in COVID-19

**Fig. 4:**
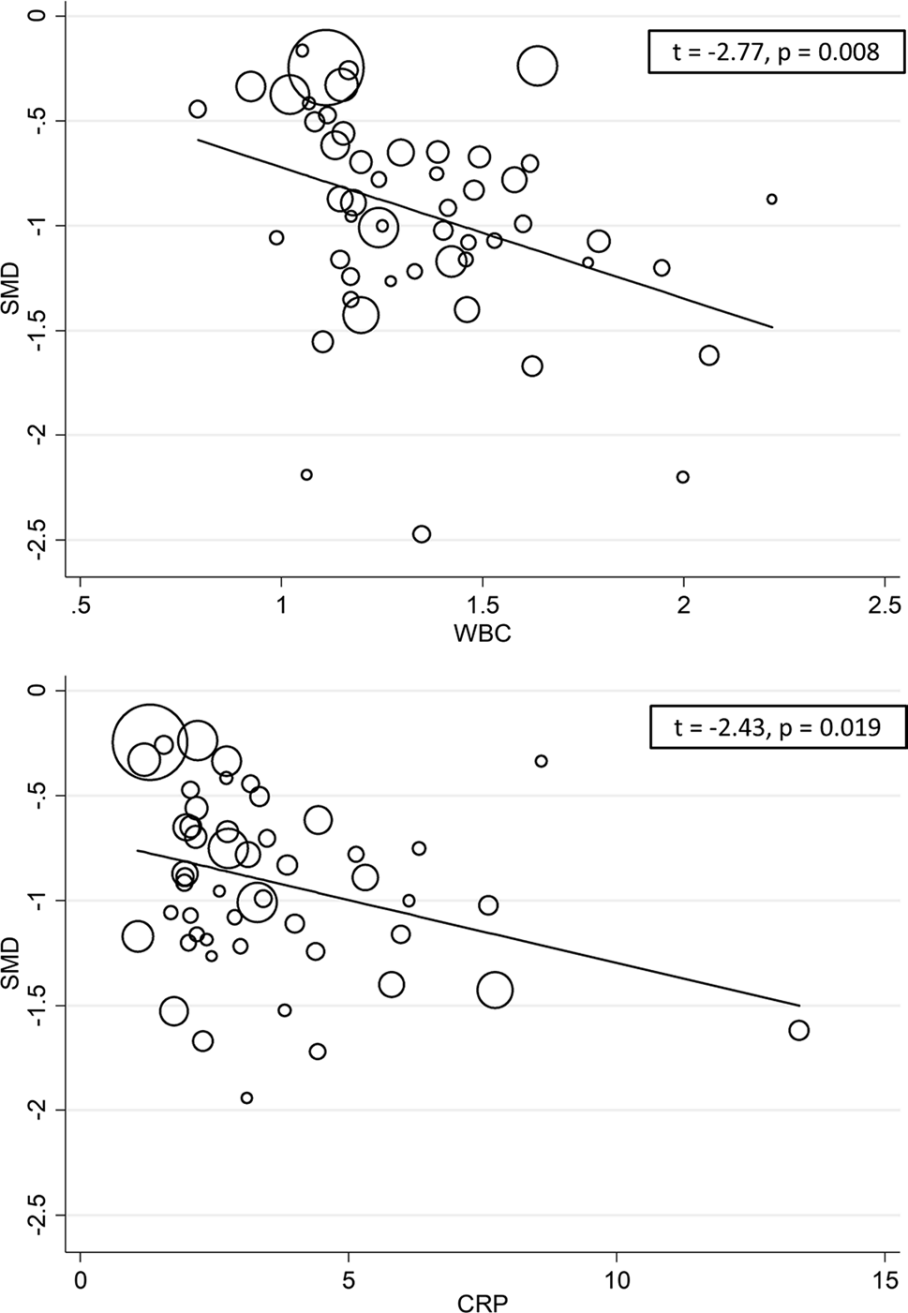
Univariate meta-regression analysis between WBC, CRP and effect size

The overall standardized mean difference in serum albumin concentrations between COVID-19 patients with low versus high severity or good versus poor outcomes in the 67 studies is shown in Fig. 2. In all studies, patients with high disease severity or poor outcome had lower albumin concentrations compared to those with low severity or good outcome (mean difference range, − 0.16 to − 2.60) although the difference was not statistically significant in four studies [28, 52, 59, 82]. The pooled results confirmed that serum albumin concentrations were significantly lower in patients with high severity or poor outcome (SMD: − 0.99; 95% CI, − 1.11 to − 0.88, *p* < 0.001). Extreme heterogeneity between studies was observed (*I*^2^ = 89.3%; *p* < 0.001). Sensitivity analysis showed that the effect size was not modified when each study was in turn removed (effect size ranged between − 0.970 and − 1.007). Evidence of publication bias was provided by a funnel plot (Egger’s test, *p* = 0.004; Begg’s test, *p* = 0.081, Fig. 3). However, trim‐and‐fill analysis showed that no study was missing or should be added. To explore possible contributors to the between-study variance, we investigated the effects of age, gender, publication geographic area, outcome, the inflammation biomarkers white blood cell (WBC) count and C-reactive protein (CRP) and the liver function markers alanine aminotransferase (ALT) and aspartate aminotransferase (AST) on SMD by univariate meta-regression analysis. Both WBC (*t* =  − 2.77, *p* = 0.008) and CRP (*t* =  − 2.43, *p* = 0.019) were significantly related to the pooled SMD (Fig. 4). In addition, the pooled SMD value in Chinese studies (− 0.99, 95% CI − 1.05 to − 0.80, *p* < 0.001; *I*^2^ = 88.2%, *p* < 0.001) was lower than that observed in non-Chinese studies (− 1.22, 95% CI − 1.43 to − 1.01, *p* < 0.001; *I*^2^ = 84.5%, *p* < 0.001) and the difference was significant by meta-regression analysis (*t* = 2.09, *p* = 0.004). No statistically significant correlation was found between SMD and age (*t* =  − 0.58, *p* = 0.56), gender (*t* = 0.46, *p* = 0.65), ALT (*t* = 0.34, *p* = 0.73) and AST (*t* = 0.40, *p* = 0.69) though a trend towards significance was observed with outcome (*t* = 1.72, *p* = 0.091). Multivariate meta-regression analysis, reported in Table 2, confirmed the significant association between effect size, WBC (*t* =  − 2.10, *p* = 0.046) and CRP (*t* =  − 2.28, *p* = 0.031) and also showed a significant relationship with age (*t* =  − 2.13, *p* = 0.043) and publication geographic area (*t* = 2.16, *p* = 0.040).

**Table 2:** Multivariate meta-regression analysis between effect size and possible contributors to heterogeneity

	*t*	*p*-value
Age	− 2.13	0.043
Gender	− 0.44	0.662
Severity/outcome	0.92	0.365
Geographic area	2.16	0.040
ALT	0.47	0.639
AST	− 0.59	0.558
WBC	− 2.10	0.046
CRP	− 2.28	0.031

## Discussion

The recently reported COVID-19 disease represents one of the worst pandemics in modern times. The disease started in China in December 2019 and spread rapidly through the globe [87]. This, the consequent lack of information regarding the pathophysiology and clinical progress, prevented the establishment and implementation of adequate public health responses. Several studies have described alterations in routine laboratory tests in patients affected by COVID-19, including a decrease in serum albumin concentrations [8–11]. However, no pooled analyses of the available evidence have been performed to accurately estimate the effect size of such reduction, to investigate its relationship with disease severity and outcomes and to evaluate which parameters may affect the effect size.

Our analysis demonstrated the presence of significantly lower serum albumin concentrations in COVID-19 patients with high disease severity or poor outcome when compared to those with low severity or good outcome. Albumin, a protein that exerts important homeostatic effects such as maintenance of the osmotic colloid pressure, intravascular transport of molecules, lipid metabolism, thrombosis and inflammation, is classically considered as a biomarker of malnutrition and poor health status [88, 89]. Hypoalbuminemia has been described as a negative prognostic factor in several diseases, [89–92]. Furthermore, low serum albumin concentrations have been shown to be associated with the severity of chronic inflammatory diseases, inflammatory bowel disease and diabetes mellitus [93], cirrhosis [94], as well as with the severity of surgical trauma [95], acute diseases [96] and sepsis [97]. In addition, in past SARS epidemics, hypoalbuminemia has been shown to be related with disease severity and increased hospital mortality [98, 99].

Extreme heterogeneity and a trend towards publication bias was observed. In multivariate meta-regression analysis, age, geographic area, WBC count and CRP were significantly associated with effect size. However, other factors not specifically investigated, such as nutritional status or assay preparation, might have also contributed to the observed heterogeneity. In particular, methodological issues with albumin determination might have influenced the heterogeneity observed between studies. It is well known that colorimetric methods for albumin determination, which are widely used in clinical institutions, are nonspecific and usually overestimate albumin concentrations when compared to the more specific and accurate immunoturbidimetric assays [100]. Unfortunately, as only few articles provided information regarding the assay used for albumin determination, we could not determine the impact of this factor on between-study variance by meta-regression analysis.

The mechanisms responsible for hypoalbuminemia in COVID‐19 have not been fully elucidated. Albumin is exclusively synthesized by the liver with a serum half‐life of approximately 21 days [101]. Notably, our analyses did not show any association between effect size and the liver function biomarkers ALT and AST, confirming previous observations that hypoalbuminemia in COVID-19 patients is not related to liver dysfunction [42]. Conversely, we found a relation between effect size and inflammation in accordance with previous studies by Huang et al. [42], which found that albumin concentrations were inversely correlated with WBC, neutrophil-to-lymphocyte ratio (NLR) and CRP, and by Huang et al. [43], that describe an inverse relationship between serum albumin and IL-6. As suggested by Huang et al. [42] hypoalbuminemia might be due to the presence of a systemic inflammatory state in COVID‐19. It is well known that inflammation may be responsible for the extravasation of serum albumin into the interstitial space due to an augmented capillary permeability, with an increased volume distribution of albumin [102]. However, it is also important to emphasize that serum albumin concentrations tend to decrease with advancing age in both sexes [103]. Therefore, the between-group differences in albumin concentrations may be in part explained by the higher disease severity and worse outcomes typically observed in older patients.

Although further research is required to investigate the relationship between albumin and COVID-19 disease outcomes, the identification of serum albumin as a marker of COVID-19 severity is biologically and clinically relevant. The determination of serum albumin concentrations, a relatively stable parameter that is strongly associated with key functional and health measures, using simple and relatively inexpensive analytical procedures, might provide rapid and useful information in regard to the overall homeostatic capacity of an individual. Consequently, the identification of relatively low serum albumin concentrations in hospitalized COVID-19 patients might assist with appropriate risk stratification and selection of suitable care pathways, even taking into consideration that age can be an important confounding factor.

In conclusion, our systematic review and meta-analysis showed that serum albumin concentrations in COVID-19 patients with high disease severity or poor outcomes are significantly lower when compared to those with milder disease. Age, geographical area and inflammation status are relevant contributors to the between-study variance. Further studies are required to investigate if albumin assessment may effectively help clinicians to early identify patients at high risk of poor outcome and if this parameter may be helpful also to successfully evaluate, at early stage, the response to treatment.
